# Impact of fish oil supplementation on plasma levels of highly unsaturated fatty acid-containing lipid classes and molecular species in American football athletes

**DOI:** 10.1186/s12986-024-00815-x

**Published:** 2024-07-08

**Authors:** Veronica Anne Mullins, Justin M. Snider, Bryce Michael, Lydia Rose Porter, Roberta Diaz Brinton, Floyd H. Chilton

**Affiliations:** 1 School of Nutritional Sciences and Wellness, Bioscience Research Laboratory (BSRL), University of Arizona, Room 370, 1230 N Cherry Avenue, Tucson, AZ 85719 USA; 2 Center for Precision Nutrition and Wellness, University of Arizona, 1230 N Cherry Avenue, Tucson, AZ 85719 USA; 3 Center for Innovation in Brain Science, The University of Arizona Health Sciences, University of Arizona, 1230 N. Cherry Avenue, Tucson, AZ 85719 USA

**Keywords:** Lipids, Docosahexaenoic acid, Eicosapentaenoic acid, Concussion, Brain, Subconcissive-impacts, Lipidomics

## Abstract

**Background:**

Previous studies have linked sports-related concussions and repeated subconcussive head impacts in contact sport athletes to elevated brain injury biomarkers. Docosahexaenoic acid (DHA), the primary omega-3 (n-3) highly unsaturated fatty acid (HUFA) in the brain, has shown neuroprotective effects in animal models after brain injury, but clinical research has shown mixed results.

**Methods:**

We conducted a randomized, double-blind, placebo-controlled study on 29 Division 1 collegiate American football players, exploring the impact of DHA (2.5 g) and eicosapentaenoic acid (EPA) (1.0 g) supplied as ethyl esters, on levels of plasma lipids shown to cross the blood-brain barrier. Dietary intake data was collected using food frequency questionnaires (FFQ). Complex lipids and unesterified fatty acids were isolated from plasma, separated via reversed-phase liquid chromatography and analyzed by targeted lipidomics analysis.

**Results:**

FFQ results indicated that participants had low dietary n-3 HUFA intake and high omega-6 (n-6):n-3 polyunsaturated fatty acids (PUFA) and HUFA ratios at baseline. After DHA + EPA supplementation, plasma lysophosphatidylcholine (LPC) containing DHA and EPA significantly increased at all timepoints (weeks 17, 21, and 26; *p* < 0.0001), surpassing placebo at Weeks 17 (*p* < 0.05) and 21 (*p* < 0.05). Phosphatidylcholine (PC) molecular species containing DHA or EPA, PC38:6 PC36:6, PC38:7, PC40:6, and PC40:8, increased significantly in the DHA + EPA treatment group at Weeks 17 (and 21. Plasma concentrations of non-esterified DHA and EPA rose post-supplementation in Weeks 17 and 21.

**Conclusions:**

This study demonstrates that n-3 HUFA supplementation, in the form of ethyl esters, increased the DHA and EPA containing plasma lipid pools the have the capacity to enrich brain lipids and the potential to mitigate the effects of sports-related concussions and repeated subconcussive head impacts.

**Trial Registration:**

All deidentified data are available at ClinicalTrials.gov #NCT0479207.

**Supplementary Information:**

The online version contains supplementary material available at 10.1186/s12986-024-00815-x.

## Background

Prior research has shown an increase in biomarkers associated with brain injury and neurodegeneration in contact sport athletes following concussions and repeated subconcussive injuries [1–8]. Animal models have demonstrated a reduction in these biomarkers when supplemented with the omega-3 (n-3) highly unsaturated fatty acids (HUFA), specifically docosahexaenoic acid (DHA) and eicosapentaenoic acid (EPA) [9–15]. However, data from clinical studies measuring the impact of DHA + EPA supplementation on biomarkers of brain injury in contact sport athletes is limited and the results are inconclusive [1, 8, 16, 17].

Supplementation with DHA, the predominant HUFA within the brain, exhibits neuroprotective effects after brain injury [18–20]. While DHA and EPA can be synthesized from the essential n-3 polyunsaturated fatty acid (PUFA), alpha-linolenic acid (ALA), this conversion is inefficient [21]. This inefficiency is due, in part, to high concentrations of dietary omega-6 (n-6) PUFA such as linoleic acids (LA) and its n-6 PUFA metabolic products that compete as substates within the HUFA biosynthetic pathway [22–25]. For example, there has been a ∼ 4-fold increase in dietary LA over the past 70 years (from 2 to 3% up to 6–9% of daily energy consumed) driven by the addition of vegetable oil products (soybean, corn, palm, and canola oils, as well as margarine and shortenings) to the modern Western diet [22, 23]. Thus, the typical US diet, including that of athletes, lacks adequate concentrations of ALA and n-3 HUFA, and this results in unbalanced n-6 to n-3 PUFA and HUFA ratios [26–29].

Numerous studies have shown increased DHA concentrations in plasma [16, 21, 30–33], inflammatory cells, and cerebrospinal fluid [34, 35] following dietary supplementation with n-3 HUFA. Notably, our research in the early 1990s revealed that human supplementation with n-3 HUFA, when complexed to triglycerides, led primarily to the incorporation of EPA into neutrophil complex lipids, namely phosphatidylcholine (PC) and phosphatidylethanolamine (PE), and this incorporation depended on duration and dose [36]. However, advancements in understanding the dynamics of incorporation of different forms of n-3 HUFAs (such as triglycerides, ethyl esters, or phospholipids) into plasma lipid classes and molecular species have been limited. This knowledge gap makes it difficult to determine the capacity of n-3 HUFA supplementation to impact cellular or brain complex lipids. Consequently, gaining a deeper understanding in this area is crucial, especially when the objective is to enrich brain lipids containing n-3 HUFA. This enrichment has the potential to benefit human health in various contexts, including recovery from concussions and repeated subconcussive injuries in contact-sport athletes [31].

DHA is not synthesized in the brain and animal models, and emerging studies suggest DHA enrichment of brain lipids occurs through two primary mechanisms. These are via: (1) passive diffusion as a non-esterified free fatty acid (FFA) or (2) an active transport system utilizing the major facilitator superfamily domain-containing protein 2 (MFSD2A) protein transporter and a DHA-containing lysophosphatidylcholine (LPC-DHA) as a substrate [37–44]. In this context, it’s essential to understand how supplementation utilizing different n-3 HUFA forms and approaches affects the composition of circulating fatty acids, including PUFA and HUFA, in plasma lipid classes and molecular species. Such insights could shed light on the inconsistent results observed across different clinical studies. In the current study, we examined the influence of n-3 HUFA, provided as ethyl esters (DHA [2.5 g] and EPA [1.0 g]), on the plasma lipid profiles in collegiate American football players.

## Methods

### Participants

National Collegiate Athletic Association Division I American football athletes, cleared by the team physician to participate in University of Arizona athletics, were recruited by research personnel as previously described [1]. A total of 38 participants volunteered and provided written informed consent. Nine participants did not complete the study protocol. There were no serious adverse events and most athletes dropped out due to unrelated injuries, time demands, or GI discomfort. A consort diagram and table of adverse events have been published previously [1].

### Dietary intervention design

**Fig. 1:**
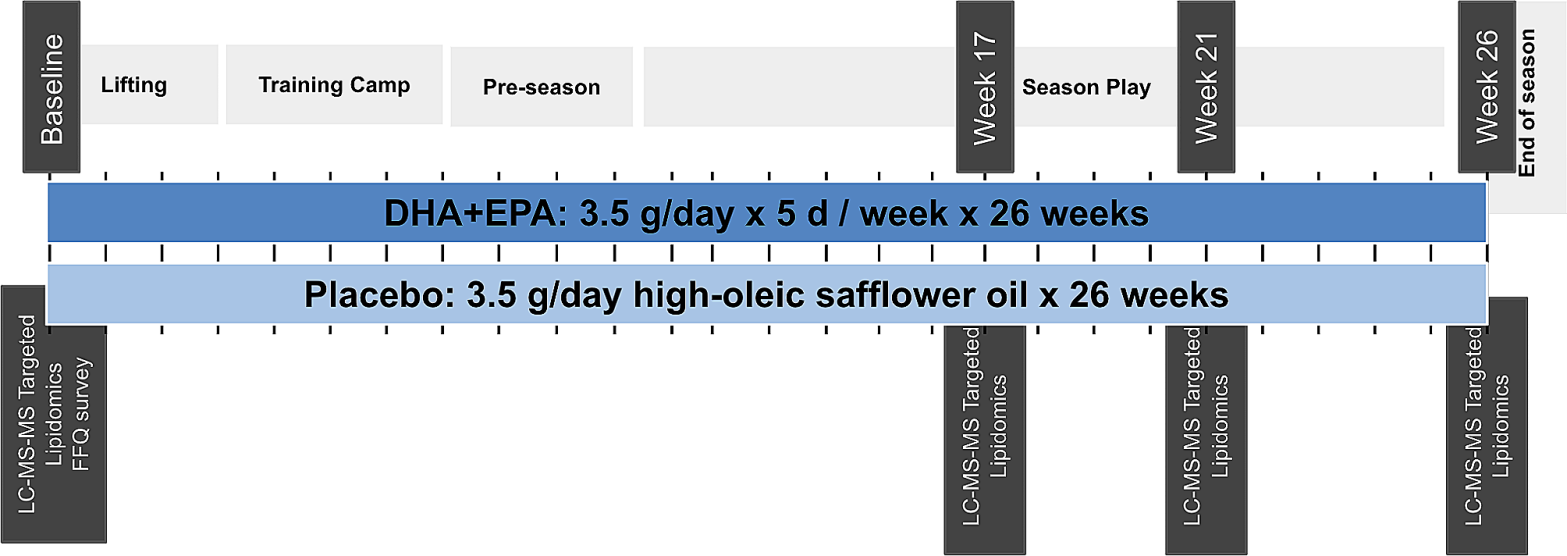
Schematic of study design

A schematic of the intervention design is illustrated in Fig. 1. A randomized, double-blind, placebo-controlled, parallel-group design was employed with DHA + EPA or placebo treatment group randomly assigned by a statistician as previously described [1]. Participants took six-1 g soft gel capsules 5-days per week for 26 weeks. In the DHA + EPA group, each capsule contained 407 mg of DHA and 170 mg of EPA as ethyl esters with the six capsules providing 2.4 g/d DHA and 1.0 g/d EPA. In the placebo group, each capsule contained no DHA or EPA but oleic acid (713 mg) and linoleic acid (14 mg) from high-oleic safflower oil with the six capsules providing 4.2 g/d oleic acid and 84 mg/d linoleic acid. Capsules, provided by Pharmavite (West Hills, California), were labeled (“A”, “B”) to maintain blinding and were identical in appearance.

The fatty acid composition of DHA + EPA and placebo capsules are described previously [1]. Supplement compliance was set at > 80% for each subject and was assessed by research staff daily via visual supervision and counting returned/unconsumed capsules. Whole blood was collected at baseline (before pre-season non-contact conditioning), Week 17 (after training camps, contact practices, and 4 regular season games), Week 21 (after 8 regular season games), and then at week 26 (3–4 days after the last game of the season) (Fig. 1).

### Dietary intake analysis

Participants completed the validated 10-page Block 2014.1 Food and Activity Questionnaire with study personnel at baseline (Block, 1990). Questionnaires were sent to NutritionQuest for analysis which provided an estimate of usual dietary intake over the previous year (NutritionQuest, Berkeley, CA). All study participants were asked to limit dietary fish and fish oil intake upon consent for the duration of the study.

### Biospecimen collection

Whole blood in 10 mL ethylene diamine tetra-acetic acid (EDTA), 7.5 mL powdered glass clot activator, and 4.5 mL tri-sodium citrate was collected by trained study personnel at each timepoint in a certified blood collection room at the football training facility. EDTA and clot activator vacutainer tubes were centrifuged at 3000 rpm for 15 min within 2 min and after 30 min of collection respectively. Aliquots of plasma from EDTA tube and serum from clot activator tube were transferred to polypropylene vials and stored at − 80 ℃ until analysis.

### Targeted lipidomic analysis to determine concentrations of phospholipids, lyso-phospholipids, non-esterified fatty acids, acyl carnitines and diacylglycerides

Lipid metabolites were isolated from plasma via a single-phase extraction method adapted from Bielawski et al. [45] and separated using reversed-phase liquid chromatography as described below. Targeted lipidomics analysis was performed using an Agilent 1200 HPLC tandem Thermo Quantum Ultra triple quadrupole mass spectrometer to quantify levels of major molecular species of lysophospholipids (LPL), phospholipids (PL), FFA, acylcarnitines (AcCa), and diacylglycerides (DAG). All data was collected and processed in Thermo LCQuan Software.

### Lysophospholipid analysis

LPL analysis was achieved utilizing mass transitions adapted from Huynh et al. [46]. 10ul of LPL internal standards mix containing 2 μm lysophosphatidylserine (LPS) 17:0(d5)/0:0, lysophosphatidylcholine (LPC) 16:0(d9)/0:0, lysophosphatidylethanolamine (LPE) 16:0(d9)/0:0 was added to samples prior to extraction and utilized for quantification of the respective LPL classes. C16, C18:1, C18:2, and C20:4 molecular species for lysophosphatidylcholine, lysophosphatidylethanolamine, and lysophosphatidylserine (Cayman Chemical) were used as standards for calibration curves which ranged from 10 pmol/ml to 5000 pmol/ml. LPLs were separated using an Agilent Poroshell 120 EC-C18 1.9 μm (2.1 × 50 mm) column with mobile phases composed of water containing 2 mM ammonium formate/0.1% formic acid (MPA) and methanol containing 1 mM ammonium formate/0.1% formic acid (MPB) at a flow rate of 300 µl/min. Chromatographic gradient elution began at 40% A and remained there for the first minute, proceeding to 1% A at 6 min and remaining there for 10.5 min, before returning to 40% MPA over 1.5 min and remaining there until the end of the 20-minute run.

### Diacylglyceride analysis

DAG analysis was achieved utilizing mass transitions adapted from Huynh et al. [46]. 50ul of DAG internal standards mix containing 1 μm DAG(19:0/0:0/19:0), DAG(15:0/0:0/15:0), DAG(16:0-d9/16:0/0:0), DAG(13:0/0:0/13:0) was added to samples prior to extraction and utilized for quantification. DAG(18:0/16:0/0:0), DAG(18:0/14:0/0:0), DAG(18:0/20:4/0:0), DG(18:2/0:0/18:2), DG(14:0/14:0/0:0), DG(24:1/0:0/24:1), DAG(16:0/0:0/18:3), DG(12:0/12:0/0:0), DG(16:0/0:0/16:0), DG(20:0/0:0/20:0), DG(18:0/20:4/0:0), DG(18:1/0:0/18:1) molecular species (Cayman Chemical) were used as standards for calibration curves which ranged from 2.5 pmole/ml to 800 pmol/ml. For molecules without standards, the closest related molecular species with a standard was utilized. DAG molecules were separated using a Peeke Spectra C-8 3 μm (3 × 150 mm) column utilizing MPA and MPB at a flow rate of 500 µl/min. Chromatographic gradient elution began at 30% A and remained there for the first minute, proceeding to 1% A at 10 min and remaining there for 9 min, before returning to 30% MPA over 0.5 min and remaining there until the end of the 30-minute run.

### Acylcarnitines analysis

AcCa analysis was achieved utilizing mass transitions adapted from Giesbertz et al. [47]. 50ul of AcCa internal standards mix containing 2 μm DL-Carnitine-d9, AcCa 10:0-d3, and AcCa 18:1-d3 was added to samples prior to extraction and utilized for quantification. L-Carnitine, AcCa 16:0, AcCa 18:2, AcCa 18:1, AcCa 6:0, AcCa 18:0, AcCa 14:0, AcCa 4:0, and AcCa 20:4 molecular species (Cayman Chemical) were used as standards for calibration curves which ranged from 2.5 pmol/ml to 800 pmol/ml. For molecules without standards, the closest larger carbon chain length standard was utilized. AcCa molecules were separated using a Peeke Spectra C-8 3 μm (3 × 150 mm) column utilizing MPA and MPB at a flow rate of 500 µl/min. Chromatographic gradient elution began at 60% A and remained there for two minutes, proceeding to 2% A at 10 min and remaining there for 7 min, before returning to 60% MPA over 0.5 min and remaining there until the end of the 20-minute run.

### Phospholipids analysis

PL analysis was achieved utilizing mass transitions adapted from Huynh et al. [46]. 50ul of PL internal standards mix containing 2 μm PC(16:0d-9/16:0), PE(16:0d-9/16:0), phosphatidylserine (PS)(16:0d-9/16:0) was added to samples prior to extraction and utilized for quantification of the respective PL classes. PE(16:0/18:1), PC(18:1(9Z)/16:0), and PS(18:1/18:1) molecular species (Cayman Chemical) were used as standards for calibration curves for their respective classes. Calibration curves ranged from 10 pmol/ml to 5000 pmol/ml. The chromatographic gradient was similar to the DAG method, though mobile phases were adapted for PL. Mobile phase A consisted of water: methanol: acetonitrile (1:1:1) with 0.1% acetic acid, while mobile phase B consisted of isopropanol with 0.1% acetic acid at a flow rate of 500 µl/min on a Peeke Spectra C-8 3 μm (3 × 150 mm) column.

Plasma phospholipid molecular species (PC, PE, PS) concentrations were assessed and putative fatty acid identification at the sn-1 and sn-2 position of the glycerol backbone was determined using PubChem (National Library of Medicine, National Center for Biotechnology Information) and Chem Spider (Royal Society of Chemistry, 2023).

### Free fatty acid analysis

FFA were extracted separately utilizing methods adapted from Okahashi et al. [48]. Extraction utilized Monospin C18 (GL Sciences) spin columns that were conditioned with methanol and water prior to sample addition. Samples were prepared by adding 50 µl MeOH (0.1% formic acid), followed by centrifugation to separate phases. The upper layer was then added to columns with 50ul of internal standard mix containing 2 μm C22:6 n-3-d5, C18:1(9Z)-d17, C20:4-d11, C16:0-d5 FFA (Cayman Chemical) and columns were washed with 40% MeOH twice prior to elution with 100 µl of 90% MeOH with 2% acetic acid. Samples were then transferred to liquid chromatography-mass spectrometry (LC-MS) vials for analysis. C14:0, C16:0, C18:0, C18:1n9, C18:2n6, C18:3n3, C20:2Δ11,14, C20:3n6, C20:4n6, C20:5n3, C22:5n3, and C22:6n3 molecular species (Cayman Chemical) were used as standards for calibration curves that ranged from 10 pmol/ml to 5000 pmol/ml. For molecules without standards, the closest larger carbon chain length standard was utilized. Gradient elution was achieved using a Peeke Spectra C-8 3 μm (3 × 150 mm) column with mobile phase A consisting of water: methanol: acetonitrile (1:1:1) with 0.1% acetic acid, while mobile phase B consisting of isopropanol with 0.1% acetic acid. Mass transitions for free fatty acid molecular species were adapted from Huynh et al. [46].

### Statistical analysis

Changes in lipid species concentrations from baseline within treatment groups and between treatment groups were compared using Student’s T-test. Other statistical analyses were performed using the RStudio statistical computing environment (RStudio, Version 1.3.1093 © 2009–2020 RStudio, PBC, Boston, MA) together with R version 4.0.5 (R: A Language and Environment for Statistical Computing, R Core Team, R Foundation for Statistical Computing, Vienna, Austria, 2020). Positively skewed outcome variables were transformed using the natural log. Linear mixed-effects models were fit using the lme4 [49] package. Hypothesis tests and estimated marginal means calculations were performed using the lmetest [50] and emmeans [51] packages, respectively. The effects of DHA + EPA treatment was compared to placebo on markers of interest (LPL, PL, FFA, AcCa, DAG) by time, group, and time-by-group interactions. In the estimated marginal means analyses, *p*-values were adjusted using Tukey’s method. *P*-values < 0.05 were considered statistically significant for all analyses.

## Results

### Participant demographic and dietary lipid intake data

Twenty-nine NCAA American football players completed a randomized, placebo-controlled, double-blinded, parallel group design trial designed to determine the impact of DHA + EPA supplementation on selected plasma lipid classes and molecular species. The complete study protocol and adverse events were reported previously [1]. Demographic data is shown in Table 1. Dietary n-3 PUFA and HUFA intake (grams) was assessed by food frequency questionnaire (FFQ) at baseline and is reported in Table 1. Unexpectedly, dietary intakes of EPA (C20:5n-3), docosapentaenoic acid (DPA)(C22:5n-3), and DHA (C22:6n-3) were significantly higher in the placebo group compared to the DHA + EPA treatment group, although these increases were ​​very modest ranging from a 0.01% increase in DPA to a 0.7% increase in DHA.

**Table 1:** Demographic characteristics, dietary n-3 fatty acid intake by group as percent of total in the placebo and DHA + EPA treatment groups

	Placebo (*N* = 17)	DHA + EPA (*N* = 12)	*p*-value
**Demographic Data** (mean [SD])			
Age (years)	20.5 (2.2)	20.3 (0.78)	0.818
Height (inches)	73.7 (2.0)	72.2 (3.6)	0.236
Weight (pounds)	221.5 (41.4)	206.8 (39.0)	0.337
**n-3 PUFA and HUFA** (mean g / d [SD]) assessed by FFQ			
C18:3	1.99 (0.69)	2.07 (1.38)	0.859
C18:4	0.02 (0.02)	0.03 (0.02)	0.697
C20:4	0.24 (0.12)	0.19 (0.10)	0.263
C20:5 (EPA)	0.06 (0.04)	0.02 (0.01)	0.004**
C22:5 (DPA)	0.03 (0.01)	0.02 (0.01)	0.035*
C22:6 (DHA)	0.13 (0.08)	0.05 (0.03)	0.003**

2-tailed, 2-sample unequal variance T-test was used for calculating *p*-value. SD, standard deviation; g, grams; d, day; n-3, omega-3; PUFA, polyunsaturated; HUFA, highly unsaturated fatty acid; EPA, eicosapentaenoic acid; DPA, docosapentaenoic acid; DHA, docosahexaenoic acid

### Effects of DHA + EPA supplementation on the concentrations of DHA, DPA, and EPA in plasma lipid classes

**Fig. 2:**
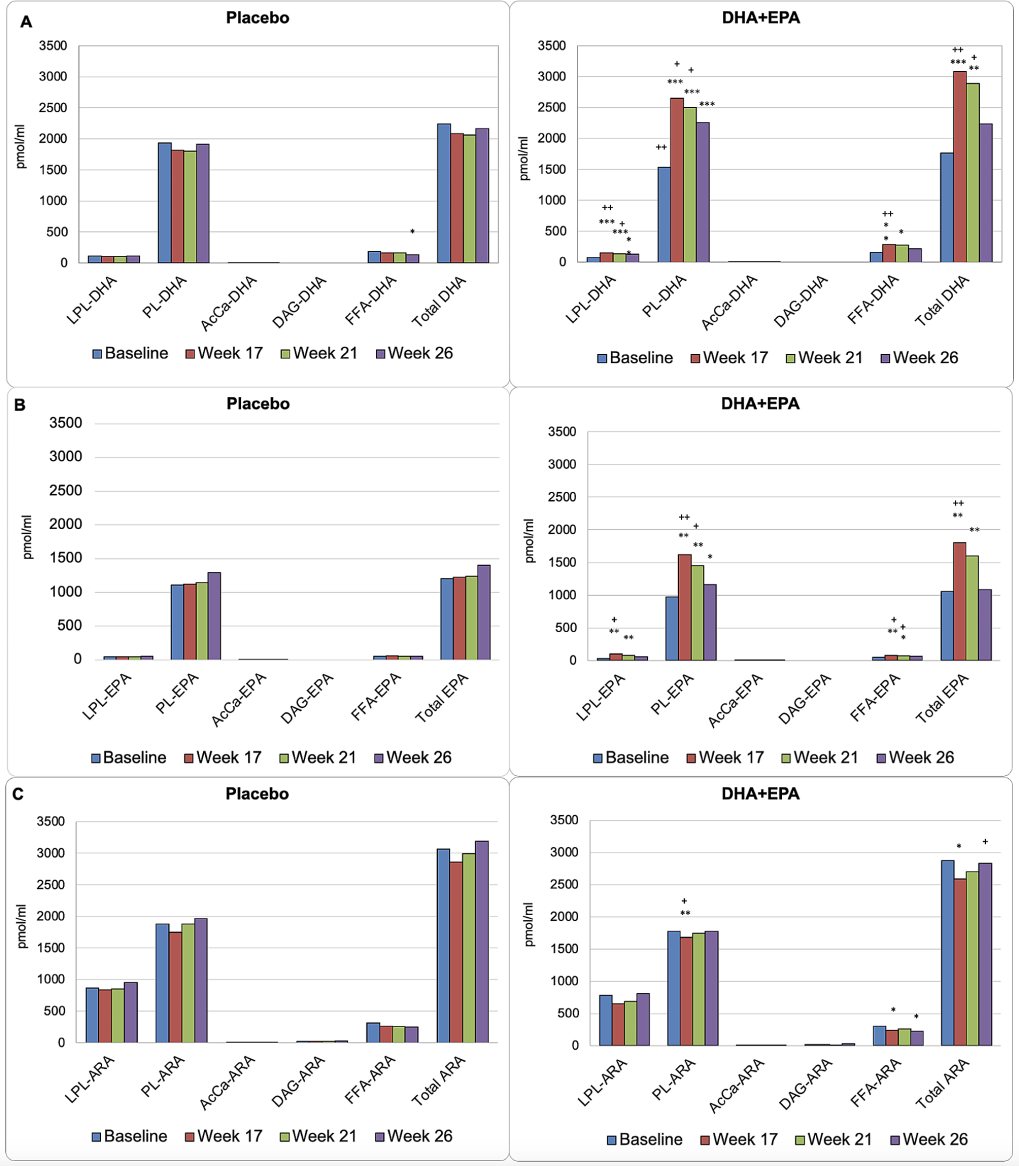
Changes in plasma concentrations of DHA (**A**), EPA (**B**), and ARA (**C**) containing lipid classes. Changes from baseline * <0.05, ** <0.01, *** <0.001, **** <0.0001. Between group differences + < 0.05, ++ <0.01, +++ <0.001. LPL, lysophospholipid; PL, phospholipid; AcCa, acylcarnitine; DAG, diacylglycerol; FFA, free fatty acid; DHA, docosahexaenoic acids; EPA, eicosapentaenoic acid; ARA, arachidonic acid

Figure 2 illustrates plasma concentrations (pmol/mL) of HUFA (DHA, EPA, and arachidonic acid [ARA]) esterified in LPL, PL, AcCa, DAG lipid classes, and FFA at baseline, Week 17, Week 21, and Week 26 following a 26-weeks regimen of placebo or DHA + EPA supplementation. Mean and standard deviation values can be found in Supplemental Table 1. In both groups (placebo and DHA + EPA), DHA was found primarily complexed to PL (PL-DHA) with smaller quantities in LPL (LPL-DHA) or as FFA (FFA-DHA) (Fig. 2A). Following DHA + EPA supplementation, PL-DHA, LPL-DHA, and FFA-DHA plasma concentrations significantly increased from baseline in the treatment group and were significantly greater than those in the placebo group (Fig. 2A). The greatest increase in DHA after DHA + EPA supplementation occurred within DHA-containing phospholipids with smaller increases in LPL-DHA and FFA-DHA.

Similar to DHA, plasma EPA was primarily found esterified within phospholipids (PL-EPA), with small quantities present complexed to LPL (LPL-EPA) or as a FFA (FFA-EPA) in baseline samples from both the placebo and DHA + EPA treatment groups (Fig. 2B). Following DHA + EPA supplementation, EPA plasma concentrations were significantly increased in PL, LPL and FFA with the greatest increase occurring in EPA-containing PL.

In both groups, ARA was primarily esterified within PL (PL-ARA) and LPL (LPL-ARA) with small quantities present as FFA (FFA-ARA) and DAG (DAG-ARA) (Fig. 2C). Following DHA + EPA supplementation, plasma concentrations of ARA within PL and FFA significantly, albeit modestly, decreased from baseline (Fig. 2C). There was no change in PL-ARA and DAG-ARA plasma concentrations in the DHA + EPA treatment group from baseline or compared to placebo (Fig. 2C).

DHA, EPA, and ARA esterified in AcCa was minimal at 1.0 pmol/mL in both treatment and placebo groups at baseline. Following DHA + EPA supplementation, DHA concentrations in AcCa (AcCa-DHA) were significantly increased and ARA concentrations in AcCa (AcCa-ARA) were significantly decreased from baseline in the supplementation group compared to placebo (Supplemental Fig. 1).

### Effects of DHA + EPA supplementation on plasma concentrations of HUFA-containing LPL classes

**Fig. 3:**
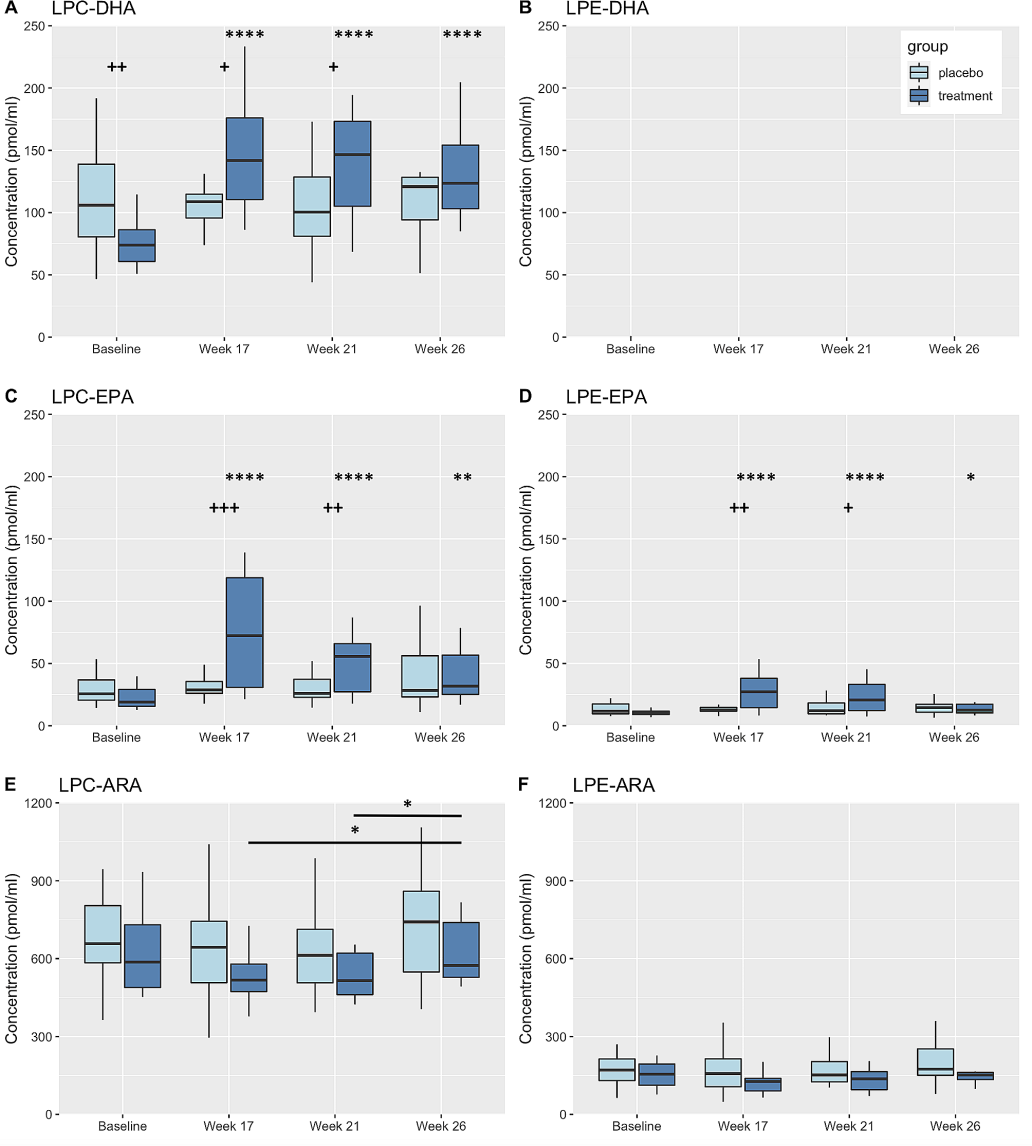
Box plots displaying changes in plasma concentrations of LPL complexed to DHA (**A-B**), EPA (**C-D**), and ARA (**E-F**). Changes from baseline and/or within group differences * <0.05, ** <0.01, *** <0.001, **** <0.0001. Between group differences + < 0.05, ++ <0.01, +++ <0.001. LPC, lysophosphatidylcholine; LPE, lysophosphatidylethanolamine; DHA, docosahexaenoic acids; EPA, eicosapentaenoic acid; ARA, arachidonic acid

As mentioned previously, DHA-containing lysophosphatidylcholine (LPC-DHA) is thought to be the primary substrate for the MFSD2A transporter so we next determined the impact of EPA + DHA supplementation on the major plasma LPL classes. At baseline in both the placebo and treatment groups, all three primary n-3 and n-6 HUFA, DHA, EPA, and ARA, were found esterified primarily within LPC compared to lyso-PE (Fig. 3).

The plasma concentration (pmol/ml) of DHA-containing LPC (LPC-DHA) was greater in the placebo group compared to the DHA + EPA group at baseline, consistent with differences in dietary intake of DHA between groups (Table 1). However, after DHA + EPA supplementation, the plasma concentration of LPC-DHA was significantly increased from baseline at all timepoints and was significantly greater than placebo at Week 17 and Week 21 (Fig. 3A). In contrast, DHA was not found incorporated into major LPE (LPE-DHA) in the placebo group or following supplementation.

The plasma concentrations (pmol/ml) of EPA within LPC (LPC-EPA) and LPE (LPE-EPA) were significantly increased in the DHA + EPA treatment group when compared to baseline at all time points. Additionally, these were significantly greater than the placebo group at Week 17 and Week 21 (Fig. 3C).

Overall, baseline concentrations of ARA-containing LPC and LPE were greater than that of DHA- and EPA-containing LPL. ARA-containing lyso-PC (LPC-ARA) was found in higher concentrations than lyso-PE (LPE-ARA) (Fig. 3E-F). The plasma concentration (pmol/ml) of LPC-ARA was not significantly different between treatment groups at any time point; however, concentrations of LPC-ARA were significantly lower in the DHA + EPA supplementation group at Week 17 and Week 21 when compared to baseline (Fig. 3E). Smaller concentrations of ARA were found in LPE molecular species compared to LPC with no between or within group differences (Fig. 3F).

### Effects of DHA + EPA supplementation on plasma concentrations of HUFA-containing phospholipid (PL) molecular species

**Fig. 4:**
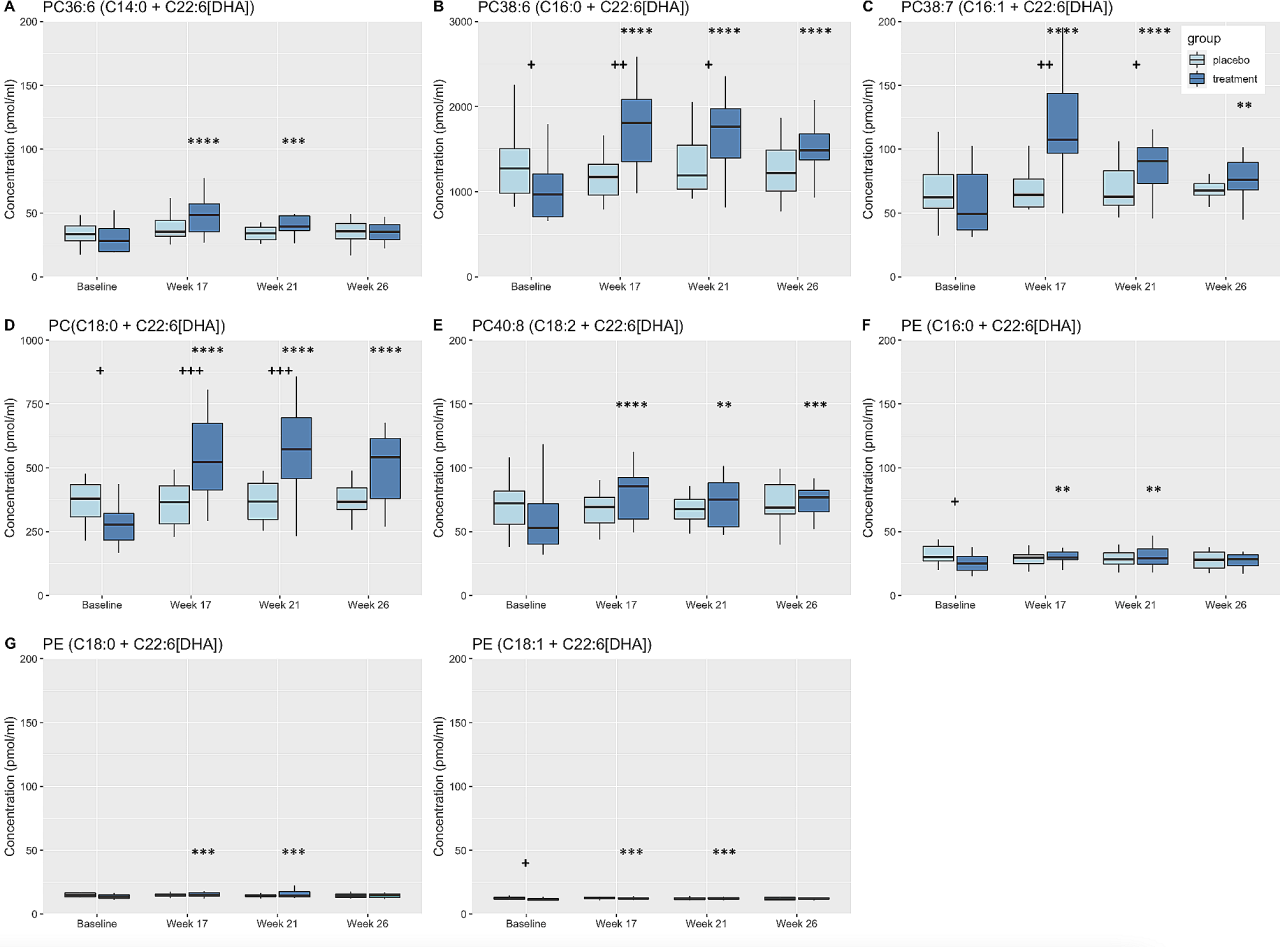
Box plots displaying changes in plasma concentrations of PL complexed to DHA. Changes from baseline and/or within group differences * <0.05, ** <0.01, *** <0.001, **** <0.0001. Between group differences + < 0.05, ++ <0.01, +++ <0.001. PC, phosphatidylcholine; PE, phosphatidylethanolamine; DHA, docosahexaenoic acids

**Fig. 5:**
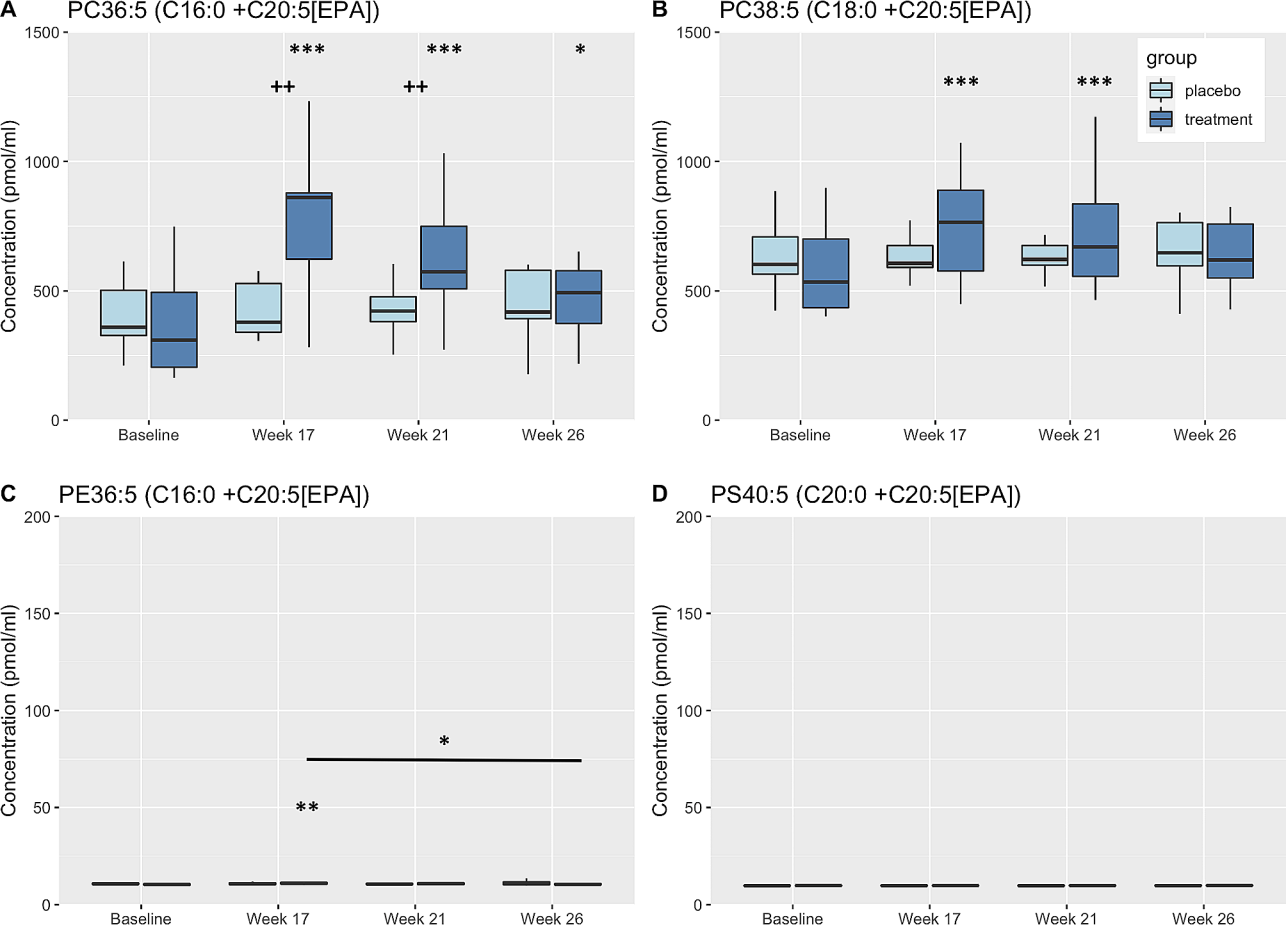
Box plots displaying changes in plasma concentrations of PL complexed to EPA. Changes from baseline and/or within group differences * <0.05, ** <0.01, *** <0.001, **** <0.0001. Between group differences + < 0.05, ++ <0.01, +++ <0.001. PC, phosphatidylcholine; PE, phosphatidylethanolamine; PS, phosphatidylserine; EPA, eicosapentaenoic acids

**Fig. 6:**
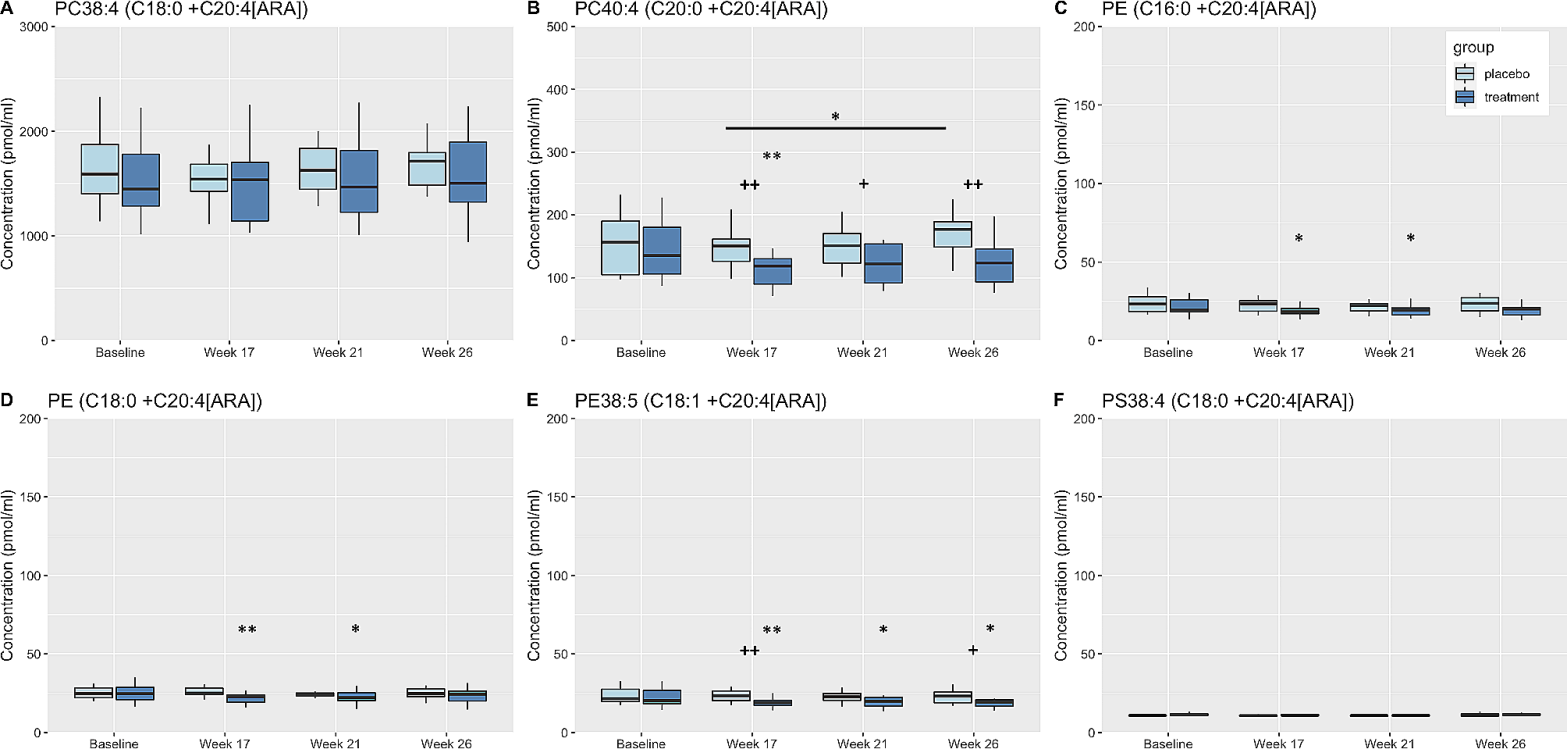
Box plots displaying changes in plasma concentrations of PL complexed to ARA. Changes from baseline and/or within group differences * <0.05, ** <0.01, *** <0.001, **** <0.0001. Between group differences + < 0.05, ++ <0.01, +++ <0.001. PC, phosphatidylcholine; PE, phosphatidylethanolamine; PS, phosphatidylserine; ARA, arachidonic acids

The primary n-3 and n-6 HUFA, DHA, EPA, and ARA, were complexed to both PC and PE molecular species, with the highest concentrations in PC (Figs. 4, 5 and 6). As expected from the class analysis (Fig. 4A), the plasma concentration of DHA-containing PC and PE molecular species increased after DHA + EPA supplementation (Fig. 4). The highest concentration of DHA in both treatment groups at baseline was found as PC38:6 (C16:0 + C22:6[DHA]) which significantly increased in the DHA + EPA treatment group compared to baseline at all timepoints and was significantly greater than placebo at Weeks 17 and 21. Plasma concentration of PC36:6 (C14:0 + C22:6[DHA]), PC38:7 (16:1 + C22:6[DHA]), PC(C18:0 + C22:6[DHA], and PC40:8(C18:2 + C22:6[DHA]) were all significantly increased in the DHA + EPA treatment group compared to baseline at week 17 and 21. In contrast, there were no temporal changes in the DHA concentrations in any of the PC molecular species in the placebo group. Overall, concentrations of DHA-containing PE molecular species were considerably less than PC; however, PE(C16:0 + C22:6[DHA]), PE(C18:0 + C22:6[DHA]), and PE(C18:1 + C22:6[DHA]) increased in the DHA + EPA group compared to baseline at Week 17 and Week 21 (Fig. 4F-H). There were no temporal changes in DHA-containing PE molecular species in the placebo group.

Like DHA, the primary EPA-containing phospholipids were PC molecular species with minimal amounts complexed in PE molecular species. PC36:5 (C16:0 + C20:5[EPA]) and PC38:5 (C18:0_C20:5[EPA]) were the primary EPA-containing PC molecular species observed and both increased significantly in the DHA + EPA group compared to baseline at weeks 17 and 21 with significant differences in the two treatment groups at those same time points for PC36:5 (C16:0 + C20:5[EPA]) (Fig. 5A-B). Plasma concentrations of PE36:5 (C16:0 + C20:5[EPA]) were minimal but did increase in the DHA + EPA treatment group at Week 17 compared to baseline and were significantly higher at Week 17 compared to Week 26 (Fig. 5C).

Plasma ARA concentrations were also higher in PC compared to PE molecular species; specifically, (PC38:4 (C18:0 + C20:4[ARA]) and (PC40:4 (C20:0 + C20:4[ARA]) were major molecular species (Fig. 6). Plasma concentrations of (PC40:4 (C20:0 + C20:4[ARA]) decreased significantly in the DHA + EPA group from baseline at Week 17 and were significantly lower than the placebo group at Weeks 17, 21, and 26 (Fig. 6B). PE(C16:0 + C20:4[ARA]) and PE(C18:0 + C20:4[ARA]) showed a significant decrease in concentration in the DHA + EPA treatment group at Weeks 17 and 21 (Fig. 6C-D). PE38:5(C18:1 + C20:4[ARA]) plasma concentrations decreased at all timepoints compared to baseline in the DHA + EPA group and were significantly less than concentrations in the placebo group at Week 17 and 21 (Fig. 6E).

### Effects of DHA + EPA supplementation on plasma concentrations of non-esterified HUFA

**Fig. 7:**
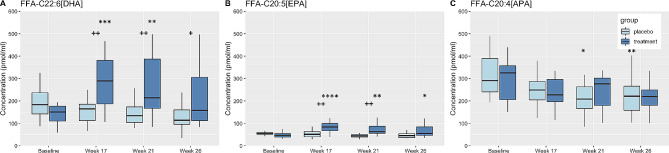
Box plots displaying changes in plasma concentrations of DHA (**A**), EPA (**B**), and ARA (**C**) as FFA. Changes from baseline and/or within group differences * <0.05, ** <0.01, *** <0.001, **** <0.0001. Between group differences + < 0.05, ++ <0.01, +++ <0.001

Plasma concentrations (pmol/ml) of DHA and EPA as FFA (FFA-DHA, FFA-EPA) increased with DHA + EPA supplementation, while there was no change in plasma concentrations of FFA containing ARA (FFA-ARA) (Fig. 7A-C). FFA-DHA plasma concentrations increased in the DHA + EPA group from baseline at Week 17 and Week 21 with significantly higher concentrations than the placebo group at all time points (Fig. 7A). FFA-EPA plasma concentrations increased from baseline at all timepoints in the DHA + EPA group, with significantly higher concentrations than the placebo group at Week 17 and Week 21 (Fig. 7B). FFA-ARA plasma concentrations decreased from baseline in the placebo group at Weeks 21 and 26 (Fig. 7C).

## Discussion

DHA is not thought to be synthesized in significant amounts within the brain [51] and therefore must cross the blood-brain barrier (BBB) from plasma sources to enrich brain tissue. Animal studies suggest two mechanisms by which DHA crosses the BBB and enters the brain: (1) through passive diffusion as FFA or (2) as DHA-containing LPC via the MFSD2A transporter [40, 41, 43, 52]. Research on HUFA incorporation of DHA and EPA into lipid molecular species following supplementation in humans remains limited. This study aimed to ascertain whether the molecular species of major polar lipid classes and FFA containing DHA and EPA were enriched when healthy athletes received supplementation of EPA and DHA in the form of ethyl esters. Such findings would shed light on whether this method of supplementation increases the lipid molecular species known to cross the BBB and thus potentially impart neuroprotection.

In this study, several significant findings emerged. Firstly, we verified both a low dietary intake of n-3 HUFA and a high n-6:n-3 essential PUFA dietary ratio of 15.5 to 1 in collegiate male athletes. This underscored the need for oral n-3 DHA + EPA supplementation to elevate plasma (and potentially brain) DHA and EPA levels. Secondly, post DHA + EPA supplementation, we observed marked increases in FFA-DHA concentrations (as shown in Fig. 7A), which are known to passively diffuse across the BBB. Additionally, we noted elevated LPC-DHA concentrations (Fig. 3A), the primary DHA molecular species proposed to be capable of crossing the BBB via the MFSD2A transporter.

Contact sport athletes are at a high risk for head injury from sports related concussions and repeated subconcussive head impacts. DHA and EPA play crucial roles in brain development and repair. Notably, DHA is present in high concentrations within neuronal cell membranes [39, 53–56]. Given the low dietary intake of n-3 PUFA and n-3 HUFA, as well as the high ratio of n-6:n-3 dietary essential PUFA [57], supplementation is necessary to elevate both plasma and brain n-3 HUFA levels. Research on animal models indicates that elevated brain n-3 HUFA concentrations, resulting from dietary DHA supplementation, can reduce neuroinflammation and enhance recovery following head injuries [10, 11, 18, 58–60]. However, there’s a major research gap in understanding the correlation between plasma and brain DHA and EPA levels in humans. Freund Levi et al. examined whether oral n-3 HUFA supplementation could alter the fatty acid concentration and HUFA profile of the cerebrospinal fluid in Alzheimer’s Disease patients (*n* = 33) [61]. Their findings highlighted an elevation in both plasma and CSF DHA, EPA, and overall n-3 HUFA concentrations in the supplement group versus the placebo group [61]. After assessing the relationship between n-3 HUFA in plasma and CSF, they found a correlation for EPA and a weaker association for DHA [61]. Here, we observed a substantial surge in circulating plasma LPC-DHA and FFA-DHA molecular species in collegiate American football athletes post-supplementation with DHA + EPA as ethyl esters. Thus, enhancing plasma levels of these DHA-containing lipids that potentially cross the BBB by known mechanisms has the capacity to elevate brain concentrations in this demographic.

Pastor et al. conducted targeted lipidomics to track the incorporation of n-3 HUFA into the plasma lipids of cystic fibrosis patients. This was a study population of 50 participants (placebo = 25, DHA = 25), who underwent 12 months of supplementation with seaweed oil at a dosage of 50 mg/kg/day, containing a maximum of 3 g of DHA/day [62]. This study found elevated n-3 HUFA plasma concentrations in the DHA-supplemented group compared to the placebo group. DHA was primarily incorporated into cholesterol esters and PC classes, which combined, accounted for 97% of the DHA increase post-supplementation [62]. In the current study which focused on lipid molecular species with the potential to cross the BBB, we observed an increase in total DHA concentrations, mainly incorporated within PC molecular species. Importantly, we also observed rises in LPC-DHA and FFA-DHA molecular species.

While this study focused on the effects of n-3 HUFA supplementation in the form of ethyl esters, supplements often present n-3 HUFAs complexed to triacylglycerides or phospholipids. Consequently, it is essential to understand not only the impact of these supplements on the levels of lipids known to cross the BBB but also to innovate new more brain bioavailable forms of n-3 HUFA-containing supplements. Such advancements could better optimize the enrichment of these plasma lipid pools for more effective brain enrichment.

## Conclusions

This study demonstrates that n-3 HUFA supplementation, in the form of ethyl esters, enhances the plasma lipid pools in male athletes, that have the capacity to potentially enrich brain lipids. Future research is needed to investigate the rise in plasma DHA resulting from various forms and doses of supplementation (ethyl ester, PL, TAG) with the objective of determining which form and dosage maximize increases in plasma DHA-LPC and DHA-FFA concentrations. Concurrently, innovations in n-3 HUFA supplement forms and strategies could be developed to optimize the enrichment of plasma lipids that traverse the BBB. Collectively, these efforts could potentially enrich brain lipids in ways that mitigate the effects of sports-related concussions and repeated subconcussive head impacts.

## Electronic supplementary material

Below is the link to the electronic supplementary material.

**Supplementary Material 1:** Box plots displaying changes in plasma concentrations of AcCa-DHA (**A**), AcCa-ARA (**B**), and DAG-[C18:0 + ARA] (**C**). Changes from baseline and/or within group differences * < 0.05, ** < 0.01, *** < 0.001, **** < 0.0001. Between group differences + < 0.05,0 ++ < 0.01, +++ < 0.001. AcCa, Acylcarnitine; DHA, Docosahexaenoic acid; ARA, Arachidonic acid; DAG, Diacylgliceride

**Supplementary Material 2**

## Data Availability

All deidentified data are available at ClinicalTrials.gov #NCT0479207.

## Abbreviations

DHA: Docosahexaenoic acid
HUFA: Highly unsaturated fatty acids
EPA: Eicosatetraenoic acid
PUFA: Polyunsaturated fatty acids
n-3: Omega-3
n-6: Omega-6
FADS1: Fatty acid desaturase 1
FADS2: Fatty acid desaturase 2
ELOVL5: Elongase 5
ELOVL2: Elongase 2
PC: Phosphocholine
PE: Phosphoethanolamine
FFA: Non-esterified free fatty acid
EDTA: Ethylene diamine tetra-acetic acid
LPL: Lysophospholipid
PL: Phospholipid
AcCa: Acylcarnitine
DAG: Diacylgliceride
LPC: Lysophosphotidylcholine
LPE: Lysophosphotidylethanolamine
LPS: Lysophosphotidylserine
FFQ: Food frequency questionnaire
DPA: Docosapentaentoic acid
ARA: Arachidonic acid
BBB: Blood-Brain Barrier
