# Supplementary figures and images for: Impact of fish oil supplementation on plasma levels of highly unsaturated fatty acid-containing lipid classes and molecular species in American football athletes
Source: Nutr Metab (Lond). 2024 Jul 8;21:43. doi: 10.1186/s12986-024-00815-x (PMC11232345; doi:10.1186/s12986-024-00815-x)

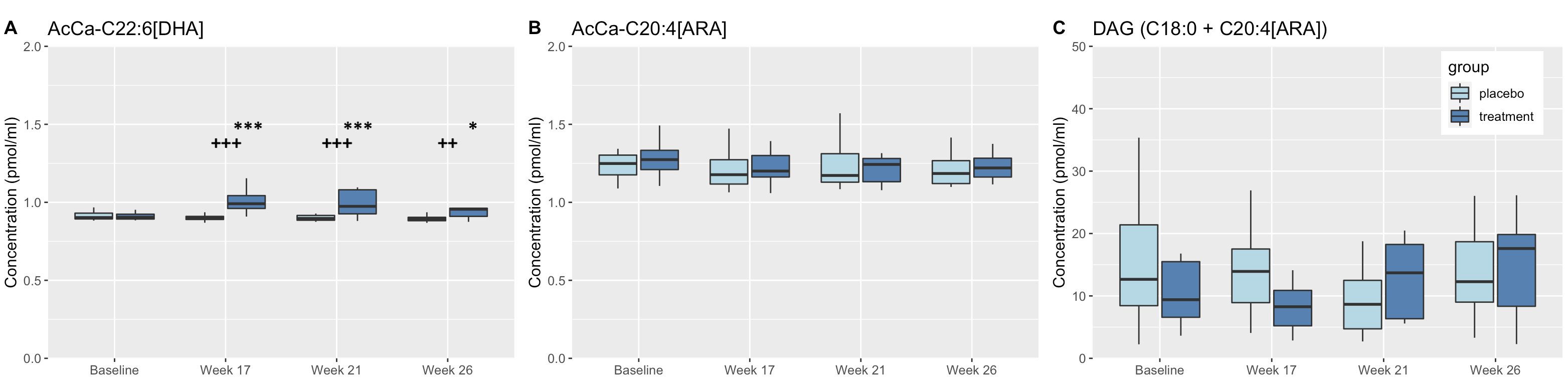

Supplement: Supplementary file 1 — Supplementary Material 1: Box plots displaying changes in plasma concentrations of AcCa-DHA (A), AcCa-ARA (B), and DAG-[C18:0 + ARA] (C). Changes from baseline and/or within group differences * < 0.05, ** < 0.01, *** < 0.001, **** < 0.0001. Between group differences + < 0.05,0 ++ < 0.01, +++ < 0.001. AcCa, Acylcarnitine; DHA, Docosahexaenoic acid; ARA, Arachidonic acid; DAG, Diacylgliceride [file 12986_2024_815_MOESM1_ESM.png]
