# Supplementary material for: Impact of fish oil supplementation on plasma levels of highly unsaturated fatty acid-containing lipid classes and molecular species in American football athletes
Source: Nutr Metab (Lond). 2024 Jul 8;21:43. doi: 10.1186/s12986-024-00815-x (PMC11232345; doi:10.1186/s12986-024-00815-x)
Supplement: Supplementary file 2 — Supplementary Material 2 [file 12986_2024_815_MOESM2_ESM.docx]

Supplemental Table 1: Mean (SD) values plasma concentrations of DHA, EPA, and ARA containing lipid classes by group and time point.

|  | **Placebo** | | | | **DHA+EPA** | | | |
| --- | --- | --- | --- | --- | --- | --- | --- | --- |
|  | **Baseline**  Mean (SD)  pmol/mL | **Week 17**  Mean (SD)  pmol/mL | **Week 21**  Mean (SD)  pmol/mL | **Week 26**  Mean (SD)  pmol/mL | **Baseline**  Mean (SD)  pmol/mL | **Week 17**  Mean (SD)  pmol/mL | **Week 21**  Mean (SD)  pmol/mL | **Week 26**  Mean (SD)  pmol/mL |
| LPL-DHA | 149.30 (46.61) | 146.12 (37.04) | 138.58 (47.26) | 156.07 (41.20) | 116.98 (26.37) | 186.66 (50.26) | 171.73 (39.33) | 175.64 (36.84) |
| PL-DHA | 1868.49 (558.36) | 1749.67 (425.50) | 1739.62 (649.83) | 1841.62 (561.95) | 1469.15 (523.82) | 2565.55 (813.25) | 2424.84 (726.92) | 1815.85 (942.55) |
| AcCa-DHA | 0.92 (0.03) | 0.91 (.03) | 0.907 (.05) | 0.90 (0.03) | 0.91 (0.02) | 1.00 (0.07) | 0.99 (0.08) | 0.96 (0.07) |
| Dag-DHA | 0.00 | 0.00 | 0.00 | 0.00 | 0.00 | 0.00 | 0.00 | 0.00 |
| FFA-DHA | 188.76 (64.17) | 161.66 (71.62) | 168.34 (119.16) | 136.63 (85.85) | 154.71 (73.17) | 286.55 (122.00) | 275.49 (149.98) | 218.88 (137.84) |
| Total-DHA | 2207.42 (623.52) | 2058.31 (455.22) | 2037.50 (726.24) | 2135.16 (629.40) | 1741.75 (575.53) | 3039.77 (938.15) | 2850.09 (869.39) | 2211.33 (1036.57) |
| LPL-EPA | 1016.30 (362.37) | 836.08 (230.47) | 900.51 (232.48) | 850.69 (244.35) | 952.62 (407.72) | 809.57 (205.27) | 904.49 (245.51) | 772.47 (187.05) |
| PL-EPA | 552.54 (287.86) | 461.61 (211.39) | 485.19 (221.36) | 444.13 (190.30) | 432.34 (291.34) | 387.36 (203.82) | 865.13 (382.41) | 722.60 (389.97) |
| AcCa-EPA | 1.26 (0.03) | 1.27 (0.03) | 1.27 (0.04) | 1.28 (0.04) | 1.27 (006) | 1.28 (0.05) | 1.28 (0.03) | 1.28 (0.05) |
| Dag-EPA | 0.00 | 0.00 | 0.00 | 0.00 | 0.00 | 0.00 | 0.00 | 0.00 |
| FFA-EPA | 51.56 (19.96) | 55.11 (11.49) | 56.05 (22.21) | 49.91 (21.65) | 66.37 (29.45) | 48.73 (12.97) | 82.48 (27.53) | 75.82 (32.52) |
| Total-EPA | 1621.58 (607.35) | 1354.00 (337.16) | 1442.95 (358.96) | 1342.99 (363.49) | 1452.59 (561.83) | 1246.94 (279.84) | 1853.38 (492.16) | 1565.84 (427.07) |
| LPL-ARA | 917.43 (223.78) | 858.92 (279.03) | 874.92 (364.82) | 993.38 (312.25) | 747.59 (199.98) | 667.67 (151.46) | 625.73 (91.54) | 802.23 (160.91) |
| PL-ARA | 1739.89 (352.93) | 1628.14 (307.94) | 1600.77 (501.33) | 1690.75 (535.97) | 1571.44 (345.19) | 1503.41 (288.72) | 1628.96 (354.29) | 1489.13 (562.55) |
| AcCa-ARA | 1.27 )0.16) | 1.23 (0.13) | 1.24 (0.15) | 1.22 (0.12) | 1.27 (0.11) | 1.20 (0.09) | 1.23 (0.13) | 1.21 (0.09) |
| Dag-ARA | 16.65 (13.24) | 22.03 (23.45) | 19.46 (22.51) | 23.76 (31.99) | 17.98 (16.98) | 12.22 (13.89) | 11.11 (12.33) | 28.14 (51.41) |
| FFA-ARA | 297.77 (104.74) | 253.51 (96.65) | 237.58 (152.65) | 229.80 (141.59) | 322.45 (74.42) | 246.18 (59.71) | 270.84 (96.98) | 249.65 (47.84) |
| Total-ARA | 2972.94 (532.60) | 2763.75 (540.65) | 2718.78 (851.90) | 2937.44 (727.48) | 2660.72 (484.79 | 2430.69 (383.34) | 2537.86 (488.49) | 2570.36 (580.60) |
